# Assessing the energy storage potential of electric hot water cylinders with stochastic model-based control

**DOI:** 10.1080/03036758.2023.2197241

**Published:** 2023-04-05

**Authors:** Baxter Williams, Daniel Bishop, Paul Docherty

**Affiliations:** a Department of Mechanical Engineering, University of Canterbury, Christchurch, New Zealand; b Department of Civil and Natural Resources Engineering, University of Canterbury, Christchurch, New Zealand

**Keywords:** Hot water cylinder, smart control, stochastic forecasting, demand side management, domestic hot water, energy storage

## Abstract

As electric hot water cylinders (HWCs) have a large capacity for thermal storage, they are well-suited for Demand Side Management (DSM). This paper compares different methods of HWC temperature control and presents a methodology to assess the amount of thermal storage available in HWCs for demand side management based on use behaviour in different household types. Simple stochastic methods for domestic hot water (DHW) demand prediction were employed to design a smart controller that produced lower rates of unmet DHW demand and higher available storage than setpoint and ripple controllers. The average storage available for DSM from the use of this smart controller is predicted to be between 3.63 and 7.20 kWh per household. These results indicate the use of HWCs for thermal storage is a low-cost viable option for peak-shaving of power system load and could decrease power system greenhouse gas (GHG) emissions in countries such as Aotearoa New Zealand, where GHG-emitting electricity generation is primarily used to meet peak loads.

## Nomenclature

$T_{HWC}$

HWC tank temperature [K]

$T_{in}$

HWC inlet water temperature [K]

$T_{amb}$

Ambient temperature [K]

$T_{min}$

Minimum HWC tank temperature [K]

$T_{max}$

Maximum HWC tank temperature [K]

$T_{set}$

Setpoint HWC tank temperature [K]

$V_{HWC}$

Volume of HWC [L]

$V_{avail}$

Volume of water available for DHW demand [L]

$P_{HWC}$

Power of water heater [W]

$P_{rated}$

Rated power of water heater [W]

$Q_{DHW}$

Heat loss from hot water use [J]

$Q_{loss}$

Heat loss from standing losses [J]

$\dot{V}$

Flow rate of water from HWC [L/s]

$K_{loss}$

Thermal loss coefficient [W/K]

$K_{mix}$

Coefficient for effects of thermostatic mixing valve

ρ

Density of water [kg/m^3^]

$C_{p}$

Specific heat of water [J/kg/K]

Δt

Timestep [s]
*N*
: Smart controller prediction horizon [hours]

## Introduction

While wind and solar electricity generation is renewable and has low Greenhouse Gas (GHG) emissions, the intermittent yield of these forms of generation is a barrier to increased adoption (Joskow 2019). Wind and solar electricity generation have large fluctuations in supply, and thus cannot always meet demand. As such, to consistently fulfil power system demand, the contribution of intermittent renewables in a power system must be limited and assisted with highly dispatchable power generation. In particular, higher reserve margins are required for power systems with a large ratio of these renewables (Madrigal and Jordan 2014).

A large share of Aotearoa New Zealand’s electricity generation capacity is from renewable resources, including 6.9% from wind turbines, which is expected to increase in the coming decades (Ministry of Business Innovation & Employment 2019a). Rapid fluctuations in wind speed can lead to intermittent generation from wind turbines, particularly due to high-speed gusts exceeding the turbines’ maximum operational speeds. Aotearoa New Zealand is an island nation and thus cannot purchase electricity from, or sell electricity to, its neighbours, so the intermittent nature of wind generation can cause undesirable spikes in the national energy supply market. Thus, intermittent renewable generation, especially in countries such as Aotearoa New Zealand where electricity cannot readily be imported/exported, requires management of electricity supply and/or demand.

There are three ways to enable greater uptake of intermittent renewable generation: (1) adding highly responsive thermal generation in the form of peaking plants; (2) adding energy storage (Zohuri 2018); and (3) increasing demand flexibility through Demand Side Management (DSM) (Denholm and Hand 2011; Lund et al. 2015).

However, peaking plants and energy storage both have limitations related to performance and cost. Peaking plants often utilise non-renewable fuels and thus have high GHG emission intensities (Edenhofer et al. 2011). Additionally, the low utilisation of peaking plants leads to a high Levelized Cost of Energy (LCOE) (Heck et al. 2016), as they require large capital expenditure per unit of electricity generated. Meanwhile, current energy storage solutions, such as pumped hydropower, compressed air, and battery storage, have low round trip efficiencies due to nested inefficiencies (Ibrahim et al. 2008), and their installation is often expensive and can have undesirable geopolitical and/or environmental impacts. For example, the cost of residential lithium-ion batteries is approximately NZD 1000–1200/kWh (Svarc 2021), and lithium mining has been shown to contribute to global warming and ozone depletion (Vandepaer et al. 2017), release toxic pollutants (Kaunda 2020), and increase opportunities for geopolitical conflicts (Altiparmak 2022; Sanchez-Lopez 2023).

By deferring electricity demand and increasing the uptake of excess power, DSM can shift the times electricity is used to suit intermittent generation and increase the integration of intermittent renewables (Gellings and Chamberlin 1987). DSM does not require the installation of additional generation or storage capacity and can typically be implemented using low-cost controllers. Thus, DSM can lower power system emissions without the capital expenditure required for peaking plants and energy storage, and with lower environmental and geopolitical consequences compared to other existing options. However, the feasibility of DSM depends on the flexibility of electricity consumption, and thus requires appliances with the ability to increase or decrease demand.

Hot water cylinders (HWCs) appear to be excellent candidates for DSM in countries such as Aotearoa New Zealand (Stephenson et al. 2018). Domestic Hot Water (DHW) heating in Aotearoa New Zealand is currently dominated by electric resistive heating, with over 85% of HWCs electrically heated (Isaacs et al. 2010). DHW energy requirements represent 27% of residential and 8.6% of national electricity consumption (Energy Efficiency and Conservation Authority 2020a). In Aotearoa New Zealand, residential HWC peak power demand is between 1.0 and 10.0 kW per HWC, with a mean peak demand of 2.2 kW (Isaacs et al. 2010). Cylinder thermal storage is also large. A typical HWC with a volume of 150 L can provide 5.2 kWh of thermal storage if heated between minimum and maximum temperatures. This storage could allow the HWC’s electrical heating load to be decoupled from DHW consumption.

The use of HWCs for DSM must balance two constraints: (1) hot water must be available for the user, which requires the cylinder temperature to be high enough to buffer upcoming demand without dropping below serviceable temperatures; and (2) to be useful for power management at times of excess power supply, the temperature of the cylinder should be kept at a minimum. As such, the energy storage available for DSM depends on hot water consumption behaviour. Since hot water demand is variable, the magnitude of energy storage available for power system management, and therefore the potential of HWCs for DSM, is not known.

Ripple control is an existing example of DSM with HWCs. Signals, ‘ripples,’ are sent to consumers’ power metres to switch off heaters during times of high demand (Energy Efficiency and Conservation Authority 2020b). While allowing some lowering of peak electricity demand, ripple control of HWCs can only delay heating, not increase it to suit times of high supply. Additionally, by switching heaters off, ripple control has no way of ensuring sufficient hot water is available to users when required.

However, different methods of water temperature control can change the energy storage potential of HWCs. HWCs are typically regulated by temperature setpoint controllers (centralheating.co.nz), which aim to keep water temperature constant. However, residential DHW demand varies throughout the day, with typical peaks in the morning and evening (Lane and Beute 1996), so setpoint control means heaters aim to keep water temperature the same during periods of high and low DHW demand, and thus contribute to daily peak electricity demand. HWC temperature can be more efficiently regulated using ‘smart controllers’ (Kepplinger et al. 2016; Booysen et al. 2019; Sonnekalb and Lucia 2019), which learn typical times of DHW demand and heat water only when demand is expected, otherwise maintaining low water temperature and increasing energy storage potential.

While it is recognised that HWCs have large thermal storage capacity and can provide demand flexibility (Stephenson et al. 2018), an accurate model is needed to determine the amount of available energy storage that can be used for DSM without compromising DHW demand fulfilment. Aspects of this question have been addressed for Aotearoa New Zealand. In Dortans et al. (2018), the national potential of HWCs for load curtailment is calculated, based on typical peak water heating loads and the total number of electric HWCs. However, the effect of this curtailment on DHW demand fulfilment is not quantified. In Razaq and Jack (2023), smart control is used to match HWC electricity consumption to intermittent solar generation without affecting demand fulfilment, but the potential for DSM with HWCs on a national scale is not quantified.

This paper presents a methodology to determine the energy storage potential of a HWC for DSM given DHW consumption, temperature constraints, and physical parameters of the HWC. Smart HWC controllers, ripple controllers, and temperature setpoint controllers are compared for available storage and unmet hot water demand (UD). Use of the smart controller is simulated in households of different sizes in Aotearoa New Zealand to determine their available energy storage potential for DSM.

## Methods

### Stochastic model of hot water demand

Predicting DHW demand can be undertaken with stochastic models (Good et al. 2015; Griffiths and Whitehouse 2017) or machine learning techniques (Gelažanskas and Gamage 2016; Lin et al. 2017; Sonnekalb and Lucia 2019). These methods allow predictions to be made on the basis of retrospective DHW demand data. Hence, a large quantity of DHW demand data is required to develop appropriate predictive models. However, collecting these data prospectively requires installation of flow metres that can be expensive when installed in sufficient quantity to accurately reflect the inter- and intra- household variability in DHW demand.

HWC electrical consumption could be used as a surrogate of DHW consumption, as many electricity retailers collect these data. However, this inference is inaccurate due to the thermal storage, mixing, and use of ripple control in HWCs that can decouple times of DHW demand from electricity consumption. Thus, these data are unsuitable for accurately inferring DHW consumption.

As original DHW demand data are hard to come by, statistical models are often used to simulate DHW demand profiles. In particular, *DHWcalc* is a programme that uses statistical models of DHW consumption to create realistic yet ‘random’ demand profiles (Jordan and Vajen 2005). Other tools are available for generating DHW demand profiles, including Booysen’s (Booysen 2021) model of DHW demand, and the LoadProfileGenerator (Pflugradt 2020) for residential electricity loads. However, DHWcalc is the most widely used tool for generating DHW demand profiles and has been used in multiple peer-reviewed studies (Kepplinger et al. 2015; Braas et al. 2020; Ochs et al. 2020; Pulkkinen and Louis 2021), so was considered the best method of generating representative DHW demand profiles.

### Hot water cylinder and controller models

HWC energy balance can be modelled with either a uniform water temperature (Kepplinger et al. 2014; Kepplinger et al. 2015; Kapsalis and Hadellis 2017; Pulkkinen and Louis 2021), a stratified tank (Jack et al. 2018), or a multi-nodal tank with varying temperature (Kepplinger et al. 2014; Pulkkinen and Louis 2021). In Aotearoa New Zealand, HWCs are required to provide water at temperatures that prevent scalding. This safety constraint is typically met by a mixing valve, which mixes water from the cylinder with cold water, proportional to the temperature of the hot water, which allows higher HWC tank temperatures without affecting safety or comfort. In this work, a thermostatic mixing valve was assumed.

For a HWC model with a uniform temperature distribution, the water temperature can be defined with Equation (1); the heat loss from hot water use defined with Equation (2); and heat loss from standing losses with Equation (3).

(1) $${\dot{T}}_{HWC}\;=\;\frac{P_{HWC}\;\text{–}\;Q_{DHW}\;\text{–}\;Q_{loss}}{\;C_{p}\;V_{HWC}}$$

(2) $$Q_{DHW}\;=\;\;K_{mix}\dot{V}C_{p}(T_{HWC}\text{–}T_{in})$$

(3) $$Q_{loss}\;=\;K_{loss}(T_{HWC}\;\text{–}\;T_{amb})$$

where *T_HWC_* is the temperature of the HWC [K]; *P_HWC_* is the power supplied by the heater element [W]; *Q_DHW_* is the heat loss from DHW use [W]; *Q_loss_* is the heat loss from standing losses [W]; is the density of water [kg/m^3^]; *C_p_* is the specific heat of water [J/kg/K]; *V_HWC_* is the volume of the HWC [L]; $\dot{V}$ is the flow rate of hot water from the HWC [L/s]; *T_in_* is the water inlet temperature [K]; *T_amb_* is the temperature of surroundings [K]; $K_{loss}$ is an empirically tuned coefficient to a first order approximation of thermal losses [W/K]; and $\;K_{mix}$ is a factor to account for a thermostatic mixing valve, and is defined by Equation (4).

(4) $$K_{mix}=\{\begin{array}{cc} \frac{T_{out}-T_{in}}{T_{HWC}-T_{in}},\; & T_{HWC}\geq T_{out}\\ 1,\; & T_{HWC}<T_{out} \end{array}$$

HWC water temperature is limited in two ways. Firstly, water must be kept below the maximum rated temperature of the cylinder (*T_max_*) to avoid overheating. Secondly, water temperature must be high enough to satisfy user requirements for hot water. As such, the water must be kept at a temperature that minimises the rate of unmet hot water demand (UD) without overheating the cylinder. In this analysis, UD is defined as the product of the change in temperature below the minimum temperature (*T_min_*), and the time for which water is drawn below the minimum temperature. As such, lower temperatures have of a higher impact on UD. UD can be defined with Equation (5).

(5) $$UD=\int_{0}^{t_{n}}H(\dot{V})H(T_{min}-T_{HWC})(T_{min}-T_{HWC})dt$$

where UD has units of K⋅mins; $t_{n}$ is the time in minutes; and H is the Heaviside function (implemented in this case withH(0)=0), which is unitless.

HWC heating is typically regulated by setpoint controllers (centralheating.co.nz), which turn the heater on if *T_HWC_* < *T_set_*, and off if *T_HWC_* > *T_set_*. Alternatively, HWC heating can be regulated by a smart controller, which uses DHW demand forecasting to determine whether to heat the tank. Smart controllers only heat the HWC when DHW demand is expected, otherwise maintaining low water temperature and increasing energy storage potential.

HWCs are not generally expected to perfectly meet DHW demand, as to do so would necessitate keeping HWCs at very high temperatures and thus greatly increase thermal loss and cost. The DHW demand that a controller aims to fulfil can be expressed: a controller that aims to meet 99% of demand would have a Demand Fulfilment Target (DFT) of 99%.

### Energy storage potential of hot water cylinders

The HWC’s Available Energy Storage (AES) for power system management is equivalent to the electrical energy required to heat the cylinder to its maximum temperature (*T_max_*) defined in Equation (6).

(6) $$AES\;\;=\;\;\;V_{HWC}\;C_{p}\;(T_{max}\text{–}\;T_{HWC})$$

HWC control involves an inherent trade-off between UD and AES, as lower UD requires higher HWC temperatures, which reduces AES. Larger HWCs lead to greater AES potential and lower UD when the same draw volumes are experienced, as increased HWC volume means more heat can be absorbed without overheating the cylinder, and a greater volume of hot water can be drawn before UD occurs.

DHW demand in developed countries is typically around 50 L per person per day (Basson 1983; Parker et al. 2015). Recommended HWC size for a dwelling depends on the number of occupants and/or the number of bedrooms. Recommended HWC sizes from HeatingForce, a United Kingdom-based organisation for the provision of heating resources, range from 120 L for a 1-bedroom house to 300+ L for a 5+-bedroom house (HeatingForce 2017 Sep 13).

### Methodology

#### Stochastic model of hot water demand

DHWcalc was used to generate two years of DHW demand data for household sizes ranging from 1 to 6 + people. These data include 10% seasonal variation, meaning DHW consumption is on average 10% higher in winter, to reflect trends in DHW consumption in Aotearoa New Zealand (Isaacs et al. 2010). One year of data were used for household-specific model training and one year for testing the controllers. The parameters for the generation of these profiles are shown in Table 1.

**Table 1.** Settings for generation of hot water demand profiles using DHWcalc. Daily draw volume was varied to reflect different household sizes – e.g. a profile for a 3-person household would have a daily draw volume of 150 L.

Parameter	Value
Daily draw volume [L/day]	50 L per person
Start day	1
Total duration [days]	730
Time step duration [s]	60
No. of categories	4
Ratio of the mean daily draw off volumes [%]	120
Seasonal variation [%]	10

Using one year from a specific DHW dataset, a stochastic model was created in MATLAB to predict future DHW demand based on time-of-day. The statistical model generated cumulative distribution functions (CDFs) for the total DHW demand over a prediction horizon (N) at each minute of the day. These distributions were used to calculate the probability of future DHW draws.

#### Hot water cylinder and controller models

At each time step, the controller calculated the volume that could be drawn from the cylinder before *T_HWC_* dropped below *T_min_*, using Equation (7). This available volume was then compared with the demand anticipated over the prediction horizon (*N*). If the predicted draw volume at the DFT probability was greater than the available volume, the controller turned the heating element on.

(7) $$V_{avail}\;\;=\;\;V_{HWC}\frac{T_{HWC}\;\text{–}\;T_{min}}{T_{HWC}\;\text{–}\;T_{in}}$$

A setpoint controller was also studied, which aimed to keep hot water temperature at *T_set_* using the algorithm shown in Equation (8).

(8) $$P_{HWC}=\{\begin{array}{cc} P_{rated},\; & T_{HWC}<T_{set}\\ 0,\; & T_{HWC}\geq T_{set} \end{array}$$

To prevent the spread of *Legionella* bacteria, HWCs in Aotearoa New Zealand are required to heat water to at least 60°C at least once per day (Ministry of Business Innovation & Employment 2019b). In the setpoint controller, this constraint was met by the setpoint temperature of 60°C. The smart controller met this constraint by heating the cylinder if the maximum temperature over the preceding 24 h was less than 60°C.

For comparison, the effect of ripple control was also investigated. As proprietary data on times when HWCs are turned off are unavailable, times of peak power demand were estimated. ‘Ripple* control’ (so designated to avoid confusion) was simulated by implementing the control logic of the setpoint controller, with no heating during hours of peak power demand. In recognition of tendencies in Aotearoa New Zealand’s electricity grid, peak hours were defined as 7am to 11am and 5pm to 9pm (Powershop 2021).

The values of HWC parameters used in this study are summarised in Table 2.

**Table 2.** Parameters used for simulation of HWCs.

Parameter	Value	Source
$T_{in}$	15 [°C]	Bulleid (2019 Apr 18)
$T_{amb}$	18.1 [°C]	Isaacs et al. (2010)
$T_{min}$	50 [°C]	Adapted from Ministry of Business Innovation & Employment (2019b)
$T_{max}$	80 [°C]	Adapted from Ministry of Business Innovation & Employment (2019b)
$T_{set}$	60.0 [°C]	Isaacs et al. (2010)
$P_{rated}$	2200 [W]	Isaacs et al. (2010)
*K*	0.854 [W/K]	Isaacs et al. (2010)
ρ	997 [kg/m^3^]	
$\;C_{p}$	4185 [J/(kgK)]	
Δt	1. [s]	

#### Energy storage potential for a standard house

To assess and compare representative AES potential of HWCs using smart, setpoint, and ripple* controllers, a standard house was defined, with occupancy, DHW consumption, and HWC volume based on average house sizes in Aotearoa New Zealand. The average number of occupants per household in Aotearoa New Zealand is 2.7 (Statistics New Zealand 2018), which is rounded to 3.0 for definition of the standard house. Average DHW consumption in developed countries is approximately 50 L per person per day (Basson 1983; Parker et al. 2015), so the standard house was defined as having average daily DHW consumption of 150 L. Industry recommendations for a 3-bedroom house are a HWC volume of 180–210 L (HeatingForce 2017 Sep 13), so the standard house was defined as having a 210 L HWC.

At each timestep, AES was calculated using Equation (6) and UD using Equation (5). The average AES, average AES during off-peak hours (‘off-peak AES’), and total UD for the testing year were calculated.

The UD of a typical setpoint controller was used as a baseline for comparison between control strategies. Total annual UD was calculated for the setpoint controller with a setpoint temperature of 60°C, the average setpoint temperature in Aotearoa New Zealand, which reflects the level of demand fulfilment deemed acceptable in practice. Thus, this level of UD was defined as an upper limit for the smart controller.

A parametric sweep was conducted to investigate the effect of smart controller parameters on HWC energy storage potential. DFT and prediction horizon N were varied to determine their effect on average AES and total UD. From this parametric sweep, the optimal combination of smart controller parameters was selected, defined as the combination of DFT and N that yielded the highest average AES without increasing total UD above the upper limit.

Results for the ripple* controller were normalised to the service error of the setpoint controller, to facilitate meaningful comparison between the controllers. A parametric sweep of setpoint temperature was conducted for the ripple* controller to find a ripple* controller with equivalent UD to that of the setpoint controller with a setpoint temperature of 60°C.

#### Energy storage potential in different households

AES potential in different households was assessed by simulating the use of the smart controller in the households shown in Table 3. Daily DHW draw volume for each household was calculated by assuming each bedroom represented an average of one person, with an average daily draw of 50 L per person per day. HWC sizes were chosen based on common industry recommendations (HeatingForce 2017 Sep 13). For each household size, the optimal combination of DFT and N was identified and used to calculate average AES.

**Table 3.** Draw volume and cylinder size for different household sizes.

Bedrooms	Daily Demand [L]	HWC Size [L]
1	50	150
2	100	180
3	150	210
4	200	250
5	250	300
6+	300	300

#### Aggregate energy storage potential in Aotearoa New Zealand

Aggregate AES potential in Aotearoa New Zealand was estimated by multiplying the AES potential from households of different sizes by the number of houses of that size in Aotearoa New Zealand. The distribution of houses with electric HWCs in Aotearoa New Zealand are shown in Table 4, where it was assumed the average of 85% electric HWCs was constant across households of different sizes (Isaacs et al. 2010).

**Table 4.** Distribution of houses with electric HWCs in Aotearoa New Zealand (Statistics New Zealand 2018).

Bedrooms	%	Number of dwellings	Dwellings with electric HWCs
1	6%	111,358	94,654
2	19%	352,633	299,738
3	44%	816,623	694,130
4	24%	445,431	378,616
5	6%	111,358	94,654
6+	1%	18,560	15,776
Total houses		1,855,962	1,577,568

## Results

### Statistical model and controller function

**Figure 1.**
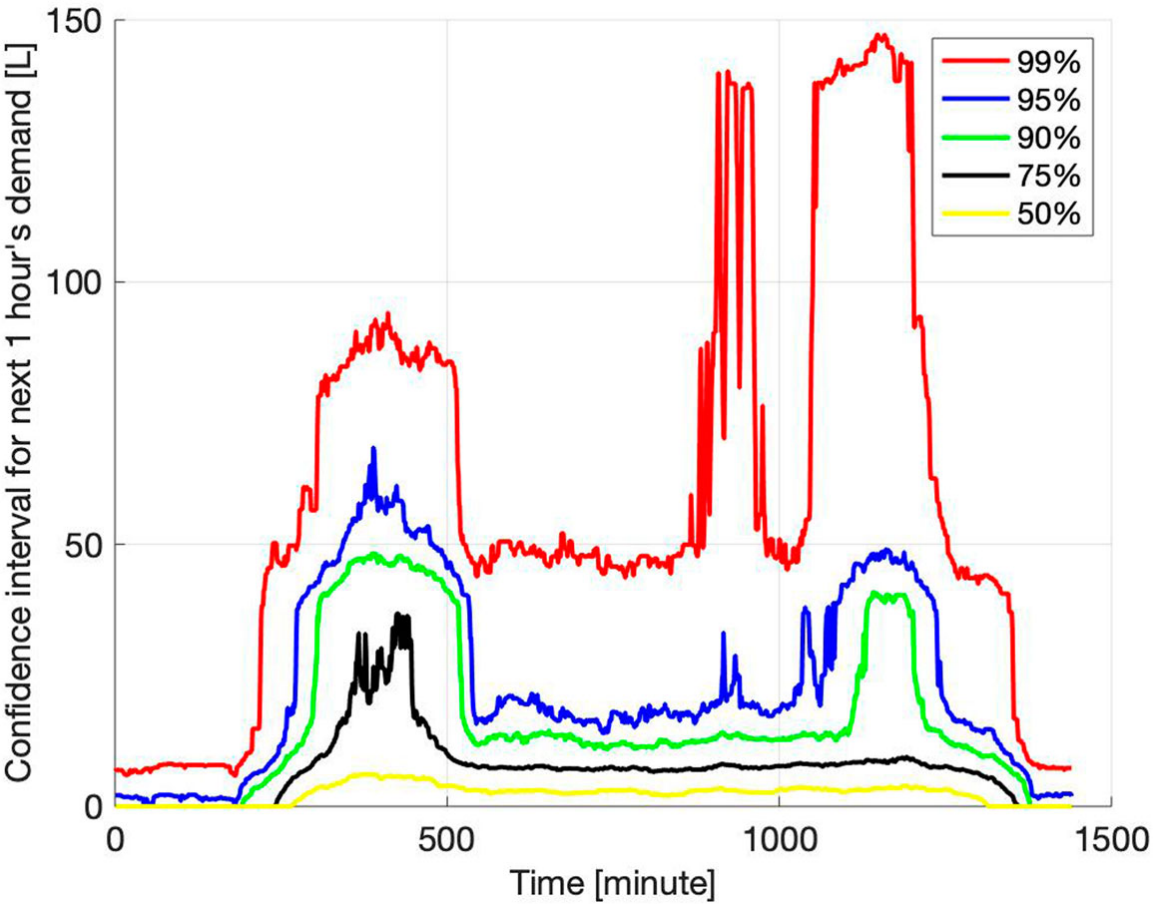
Statistical model showing predicted DHW draw volumes for a standard 3-bedroom house over the next hour throughout the day.

**Figure 2.**
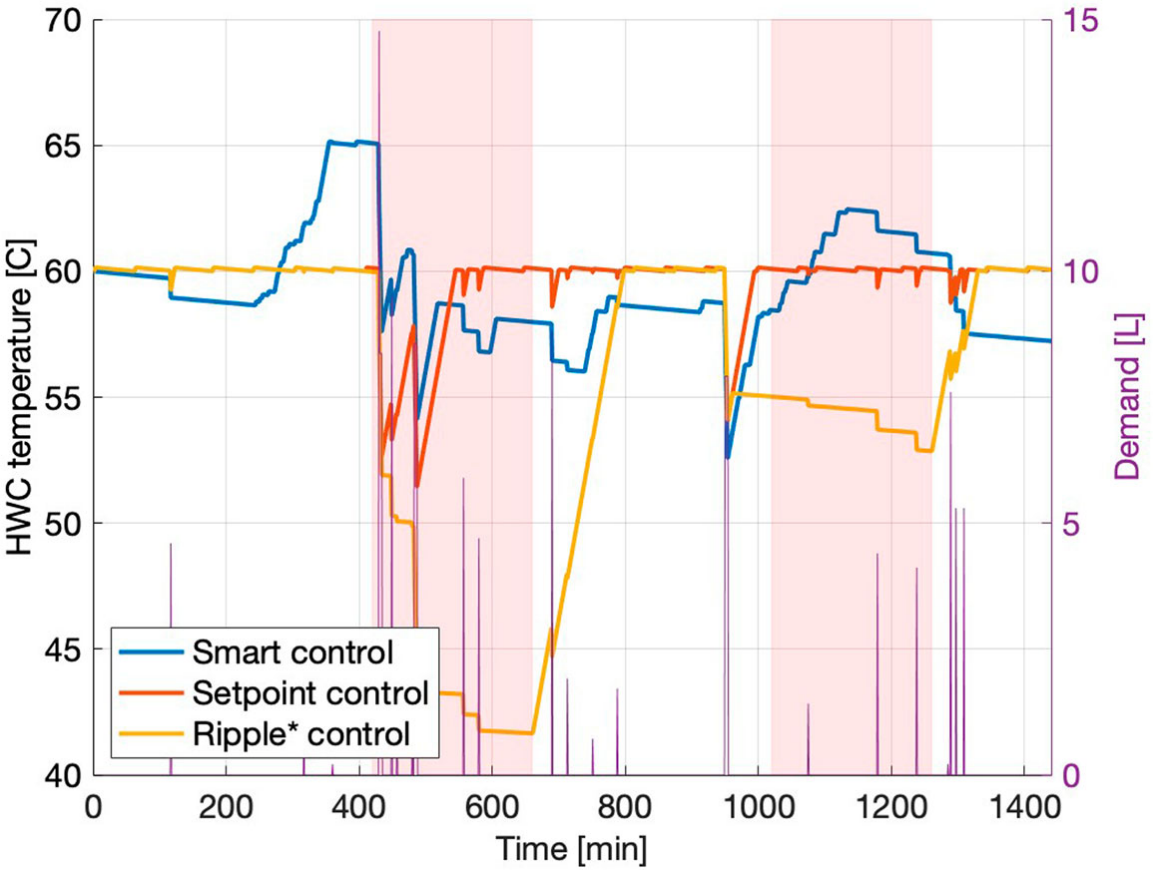
HWC Temperature from the smart, setpoint, and ripple* controllers for a representative day, with the DHW demand. Hours of peak electricity demand are shaded red to show when heating is restricted for the ripple* controller. Note that while the setpoint and ripple* controllers try to keep the temperature constant, the smart controller allows temperature to fall until it predicts future demand and raises the temperature as required.

The predicted draw volumes for one-hourly DHW demand from the one-year training period for the standard house are shown in Figure 1. The cylinder temperature from implementation of the smart, setpoint, and ripple* controllers and the DHW demand over the course of a representative day for the standard house are shown in Figure 2, with peak electricity demand times shaded red to show when the ripple* control restricts heating.

**Figure 3.**
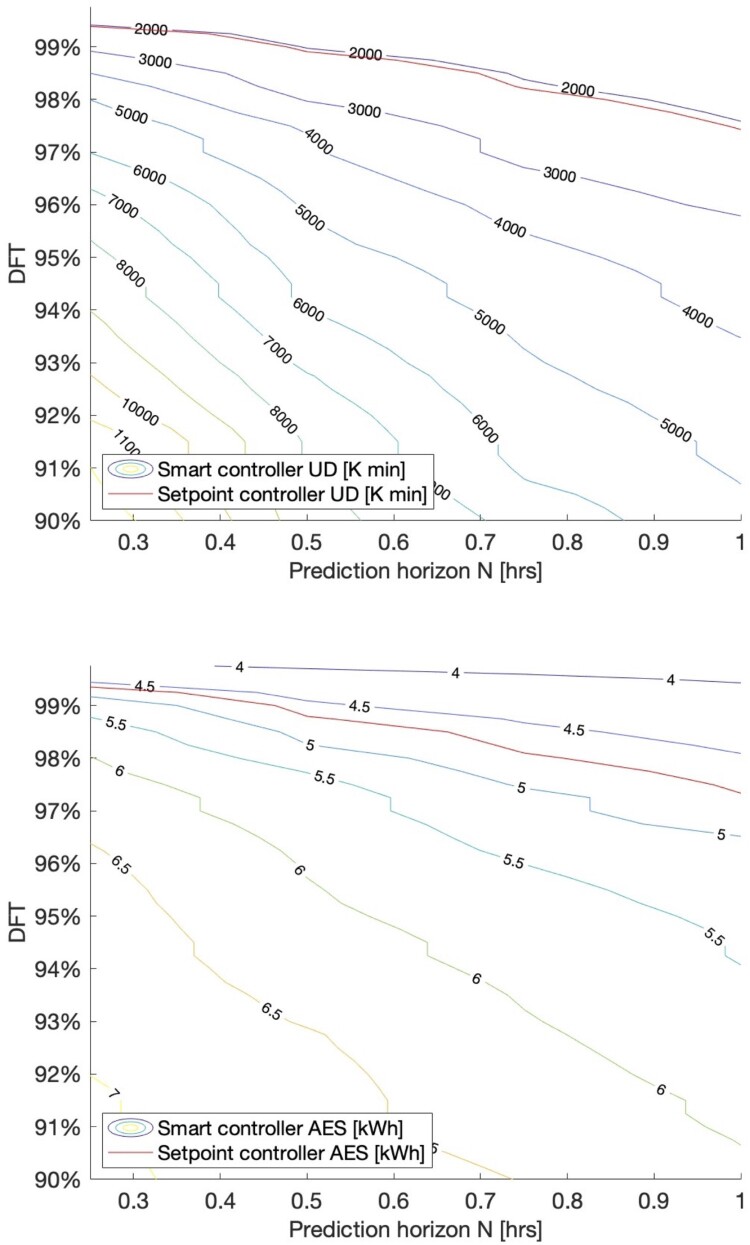
Contour plots of annual UD (top) and average AES (bottom) from a parametric sweep of DFT and prediction horizon N, for a smart controller installed in the standard house. Red lines indicate the UD and AES of the setpoint controller.

The annual UD and average AES from the parametric sweep show the optimal combination of DFT and prediction horizon *N* for the standard house (Figure 3). The optimal combination was found to be a prediction horizon of 1.0 h and DFT of 96.5%, as this combination yields the highest AES without increasing UD above that of the setpoint controller.

### Energy storage potential for a standard house

The average available AES, average off-peak AES, and annual UD from implementation of the setpoint, ripple*, and smart controllers for the standard house are shown in Table 5. If the smart controller’s combination of DFT and prediction horizon N were instead selected to match the UD of the ripple* controller, smart controller AES increased to 6.38 kWh. The ripple* setpoint temperature that gave total UD equivalent to that of the setpoint controller was 67.6°C.

**Table 5.** Setpoint, ripple*, and smart controller average AES, average off-peak AES and annual UD, with the smart controller’s optimal combination of DFT and prediction horizon, and a ripple* controller with setpoint of 67.6°C.

	Average AES [kWh]	Average off-peak AES [kWh]	Annual UD [K min]
Setpoint	4.96	4.92	2510
Ripple*	3.52	3.42	2521
Smart	5.01	5.17	2508

### Energy storage potential for different households

Average AES for each household size is shown in Table 6. Available storage was found to be higher in houses with larger HWCs, indicating these households would be the best candidates for DSM.

**Table 6.** Average storage for each kind of household, and total average storage available to the grid.

# People	Daily Draws [L]	HWC Size [L]	Average AES [kWh]
1	50	150	3.63
2	100	180	4.30
3	150	210	5.01
4	200	250	6.23
5	250	300	7.27
6+	300	300	7.20

#### Aggregate energy storage potential in Aotearoa New Zealand

Average available AES from each size of household and the total average storage available to the grid in Aotearoa New Zealand are shown in Table 7. Aggregate average AES was found to be 8.42 GWh.

**Table 7.** Aggregate average AES for households in Aotearoa New Zealand.

Bedrooms	Aggregate AES [GWh]
1	0.36
2	1.31
3	3.50
4	2.39
5	0.69
6+	0.16
Total	8.42

## Discussion

The smart controller was shown to outperform both setpoint and ripple* controllers for AES and UD, and increase the potential for DSM. Table 5 shows use of the smart controller in the standard house increases AES and decreases UD compared to both the setpoint and ripple* controllers. Additionally, off-peak AES is higher with the smart controller than with the setpoint and ripple* controllers. Thus, smart control is a better candidate than ripple* and setpoint control for DSM in the standard house, as the smart controller can heat the HWC more during off-peak electricity hours, lowering peak electricity demand. Setpoint and ripple* controllers are constrained by their inability to heat water above the setpoint temperature, so cannot implement off-peak heating like the smart controller.

HWCs were also shown to be good candidates for DSM with the smart controller in all household sizes (Table 6). Table 6 shows the predicted average AES with a smart controller is between 3.63 and 7.20 kWh per household. The likely cost for such smart controllers, when considering existing smart switches, is in the order of 100 NZD. These values indicate a nominal cost of ∼15–30 NZD/kWh, which is much lower than other existing storage methods. While DSM with HWCs is not the same as other energy storage methods as electricity cannot be discharged back into the power system, the ability to use excess electricity and therefore increase the penetration of intermittent generation at such a low cost makes this concept an attractive power system management solution.

If smart controllers were installed in all the private dwellings in Aotearoa New Zealand with electric HWCs, average aggregate AES is expected to be 8.42 GWh. Energy storage of this magnitude could greatly increase the penetration of electricity generation from intermittent renewables. Data on the presence of electric HWCs in different sizes of households are unknown, so the 8.42 GWh average AES is thus only an estimate. However, the large amount of AES calculated in this study indicates HWCs can have considerable potential for DSM in Aotearoa New Zealand.

Figure 3 shows a tradeoff between AES and UD for the smart controller. The smart controller parameters were tuned to produce similar UD levels to the setpoint controller. However, the choice of matching setpoint controller UD was somewhat arbitrary and was made only to meaningfully compare AES between controllers. With the smart controller, it is possible to alter the prediction horizon and DFT to adjust AES and UD. As such, it would be possible to increase AES further at the cost of increased UD, or vice-versa. For example, when the smart controller parameters were tuned to produce similar UD levels to the ripple* controller, AES increases to 6.38 kWh with annual UD of 6195 K*min.

Due to a scarcity of DHW demand data, or where the quality of data is reduced by metering power use rather than hot water use directly, ‘random’ DHW demand profiles were generated using ‘DHWcalc’. Real DHW data for a household are likely to be more predictable than these ‘random’ profiles. Thus, more precise stochastic models may be found with real DHW demand data. These precise DHW demand models are likely to yield more optimal cylinder control parameters, and lead to increased storage potential and lower UD with the use of smart controllers.

Additionally, the stochastic model used in this study for DHW demand prediction only includes time of day as a parameter, as the parameters of the stochastic model were limited by the ‘random’ DHW data used. Introduction of additional parameters, such as outside temperature, to the stochastic models to capture additional variability could lead to greater modelling precision. The smart controller heated the cylinder based on prediction horizon and DFT. Increasing the parameterisation of the control model or incorporating more advanced numerical methods could control the cylinder temperature more effectively and lead to greater storage and lower service errors.

The ripple* controller is an imperfect model of ripple control, as the times at which ripple switches are used to lower electricity demand depend on electricity demand at a regional or national level. Thus, simulation of actual ripple control would require the use of specific electricity demand data, which would lower the generalizability of the results. However, the ripple* controller simulated in this study used typical times of peak demand to approximate the heating constraints imposed by ripple switches, and the results for the ripple* controller should be considered indicative of those for actual ripple controllers.

The smart controller used in this study is tuned to the specified DHW demand profiles and physical HWC parameters. These parameters, such as HWC insulation and surface area, would need to be accurate to each HWC for implementation of smart control. However, the values used in this study are tuned to the national average standing losses of 2.4 kWh/day (Isaacs et al. 2010) and thus represent a standard HWC in Aotearoa New Zealand, so are sufficient for calculating national AES potential.

In this study, weather and seasonal effects were not included as variables. Colder months typically lead to higher DHW use and lower ambient temperatures. Increased DHW demand during colder months was captured in this study by the 10% seasonal variation in average daily DHW demand data. However, the effect of changes in ambient temperature on standing heat loss was not explored in this study. Instead, a constant ambient temperature of 18.1°C, the annual average ambient temperature for HWCs in Aotearoa New Zealand (Isaacs et al. 2010), was used. Results in this study are presented for average houses in Aotearoa New Zealand, which are used to calculate aggregate national average AES, so the assumption of a constant average ambient temperature limited model complexity without compromising the generalizability of results. Additionally, Aotearoa New Zealand’s electricity supply is constrained in winter months, as lower lake levels and less sun reduce electricity from hydroelectric and solar generation, respectively. However, electricity supply was not modelled in this study, so seasonal differences in electricity supply are outside the scope of this work.

This study assumed water in the HWC was fully mixed, which is a common assumption when short computational times are desired (Kepplinger et al. 2014, 2015; Kapsalis and Hadellis 2017; Pulkkinen and Louis 2021). However, thermal stratification in HWCs means the water is not fully mixed, so this simplified model can be less accurate than other models that include thermal stratification. Cold and hot water typically enter and exit HWCs at different points, so AES in stratified HWCs is likely to be higher than the values of AES calculated using a fully mixed model. Thus, the assumption of a fully mixed HWC was chosen for this study to reduce computational time and provide a conservative estimate of aggregate national AES.

The smart controller DSM ultimately led to a form of demand deferral. Power demand was shifted away from periods of high aggregate electricity demand or price. Explicit application of demand deferral was not incorporated in the approach. This implementation would require imposition of constraints such as electricity demand profiles or cost profiles and would thus require a specific application that may limit generalizability of the results. However, the smart controller was designed to optimise water heating in a stochastic environment and would therefore be capable of demand deferral, which should be explored in future work.

## Conclusion

Using statistical methods for DHW demand prediction, a smart controller was designed that out-performed a setpoint controller and ripple* controller by producing lower UD and higher average AES for DSM. Households of different sizes were predicted to have potential for 3.63–7.20 kWh of available storage, and use of all electric HWCs in Aotearoa New Zealand was estimated to give an average of 8.42 GWh of aggregate available storage. These results show inexpensive augmentations to electric HWCs are a candidate for peak-shaving of power system load. This reduction in peak demand would allow for increased penetration of electricity generation from intermittent renewable sources and decrease power system emissions.

## Abbreviations

Abbreviations: DSM: Demand Side Management; HWC: Hot Water Cylinder; DHW: Domestic Hot Water; NZ: Aotearoa New Zealand; NZD: New Zealand Dollar; GHG: Greenhouse Gas; LCOE: Levelised Cost of Energy; UD: Unmet Demand; AES: Available Energy Storage; DFT: Demand Fulfilment Target; CDF: Cumulative Distribution Function
